# Managers’ experiences of collaboration and individual participation in integrated healthcare and social services for adults with complex needs: a focus group study

**DOI:** 10.1186/s12913-026-15602-y

**Published:** 2026-09-12

**Authors:** Markus Hjelm, Veronica Plessen, Anna Andersson, Ulrika Harris

**Affiliations:** 1 Blekinge Centre of Competence, Region Blekinge, Karlskrona, Sweden; 2 Department of Clinical Sciences in Malmö, Lund University, Lund, Sweden

**Keywords:** Adults with complex needs, Collaboration, Experiences, Integrated care, Management, Multimorbidity, Participation, Psychiatric comorbidity, Qualitative research

## Abstract

**Background:**

Adults with long-term mental, cognitive, and physical conditions experience a substantial disease burden and have complex health needs. The co-occurrence of mental health problems, substance use, and social vulnerabilities further underscores the need for integrated and coordinated healthcare and social services. Such integration requires close collaboration among involved actors; however, this is often constrained by legislative barriers and challenges in coordinating complex care. Coordination adapted to complexity, individualised care planning, and interprofessional collaboration depend on effective leadership and governance to support the development and sustainability of integrated care. Therefore, this study aimed to explore managers’ experiences of facilitators and barriers to collaboration and individual participation in managing healthcare and social services for adults with complex needs.

**Methods:**

A descriptive qualitative design was employed using three focus group interviews with a total of 13 participants to explore managers’ experiences. Data were analysed using qualitative content analysis.

**Results:**

The analysis resulted in three categories: (1) organisational prerequisites for the sustainable improvement of integrated care, (2) prerequisites for interprofessional collaboration in addressing individuals’ needs, and (3) supporting individual change through relational and needs-based approaches. These categories included eight subcategories.

**Conclusions:**

Interprofessional collaboration in the care of adults with complex needs is influenced by relational, structural, and contextual factors. Although managers express awareness of and ambition towards person-centred and integrated care, a gap remains between intention and implementation. Addressing this gap may require strengthening trust, clarifying structures and governance, and supporting organisational continuity to enhance sustainable collaboration and individual participation.

**Supplementary Information:**

The online version contains supplementary material available at https://doi.org/10.1186/s12913-026-15602-y.

## Background

Adults with long-term mental, cognitive, and physical disorders often have substantial disease burden and complex health needs [1]. Individuals with severe mental illness are more than twice as likely to have multiple physical health conditions as the general population [2]. Consequently, this population frequently utilises acute healthcare services, contributing to increased healthcare costs [3, 4]. Psychiatric disorders, harmful substance use, and addiction commonly co-occur, further increasing the need for comprehensive and coordinated treatment approaches [5]. In addition, adults with complex needs are disproportionately affected by homelessness and exposure to violence and may have limited insight into their condition or be reluctant to engage in care [6]. Together, these interconnected challenges highlight the importance of integrating physical and mental health services to address multifaceted needs and improve health outcomes [1, 7].

According to the National Institute for Health and Care Excellence [8], adults with complex needs are defined as individuals aged 18 years or older who require extensive support across multiple aspects of daily life and rely on services from several health and social care providers. These needs may arise from illness, disability, broader life circumstances, or a combination of these factors [8]. In this study, the term adults with complex needs is used in line with this definition, with a particular focus on individuals with co-occurring mental health problems, substance use, and social vulnerability. Addressing the needs of this population requires coordinated and multidisciplinary approaches across healthcare and social services. In line with the SELFIE network [9], an EU research initiative focusing on integrated care for people with multimorbidity, coordination adapted to complexity, individualised care planning, and interprofessional collaboration are central components of integrated care. The implementation and sustainability of these components depend on effective leadership and governance across organizational boundaries.

Integrated care is widely recognised as essential for addressing the needs of adults with complex needs. However, healthcare systems designed to support this population are often difficult for both individuals and professionals to navigate, which may lead to unmet care and support needs [10, 11]. Studies have shown that integrated care, characterised by collaboration, patient participation, a holistic perspective, and continuity, can improve care for this population [12, 13]. Nevertheless, many healthcare systems remain organised around single conditions rather than multiple co-occurring conditions, contributing to fragmented care and an increased treatment burden. Consequently, person-centred care and interprofessional collaboration between healthcare and social services have been emphasised [14]. However, effective collaboration between providers remains a challenge in practice [6].

Leadership in integrated care is inherently complex, as collaboration often spans multiple organisations operating under different mandates, governance structures, and legislative frameworks [9]. Clear governance structures and transparent decision-making processes are important for clarifying the roles and responsibilities of integrated care [9, 15, 16]. When governance and accountability are unclear, responsibility for collaboration may weaken, potentially hindering the development and sustainability of integrated care. In such contexts, leadership is often shared across organizational boundaries and requires coordination, trust, and alignment between services and professional groups [16].

In Sweden, national initiatives aim to improve healthcare and social services for individuals with co-occurring substance use and psychiatric disorders, conditions that require coordinated interventions across sectors, including addiction treatment [17]. Further research is required to understand how managers experience and navigate collaborative practices when working with adults with complex needs. Therefore, this study aimed to explore managers’ experiences of facilitators and barriers to collaboration and individual participation in managing healthcare and social services for adults with complex needs.

## Methods

### Design

A descriptive qualitative design [18] was applied using focus group interviews [19] to explore participants’ experiences. This approach allows participants to interact with and reflect on shared experiences, which may enrich the data. The interview data were analysed using qualitative content analysis [20, 21].

### Setting

This study was conducted in a region in southern Sweden and its surrounding municipalities. In this context, responsibility for healthcare and social services for adults with complex needs is shared between regional and municipal authorities [22]. Municipalities are responsible for providing support and care for individuals with substance use disorders in accordance with the Social Services Act [23]. This includes ensuring access to appropriate care and support, as well as social welfare services such as housing support, employment initiatives, financial assistance, and support for children, relatives, and significant others. Regional authorities are responsible for providing medical care to all residents under the Health and Medical Services Act [24]. This includes medical and psychiatric interventions, treatment of withdrawal symptoms, and medication-assisted treatment of alcohol and drug dependence. In cases of severe substance use, municipalities may apply for compulsory care under the Care of Substance Abusers Act [25]. Individuals may also receive compulsory psychiatric care under the Compulsory Psychiatric Care Act [26]. Overall, the responsibility for adults with co-occurring disorders, in which substance use disorders coincide with psychiatric conditions, is distributed across several authorities. Therefore, both regional and municipal organisations play important roles in providing coordinated care and support.

### Participants and data collection

Participants were recruited through purposive sampling [27] to include managers in healthcare and social services who could provide rich descriptions relevant to the study’s aim. The inclusion criteria were as follows: (i) employment in a leadership role within healthcare or social services in regional or municipal services, and (ii) experience of leading services that work with adults with complex needs. As these individuals often require coordinated interventions from both municipal and regional services, participants also needed experience in inter-organizational collaboration. Eligible leadership roles included positions such as operational managers, roles with medical responsibility for nursing or rehabilitation, and quality or care development coordinators. Recruitment aimed to ensure variation in professional roles and organizational affiliation (regional and municipal) to capture diverse perspectives relevant to the study’s aim.

An invitation to participate was distributed via email to an established regional collaboration group working with adults with complex health needs, together with an information letter. The research project and conditions for participation were also presented verbally to the group, allowing potential participants to ask questions or contact the research team subsequently. Individuals who expressed interest received additional information and were informed that participation was voluntary and that they could withdraw at any time without providing a reason. In total, 16 participants initially agreed to participate; however, three later withdrew due to illness or competing work priorities. All participants who consented to participate provided written informed consent prior to data collection.

In October 2023, three focus group interviews were conducted in person, comprising three, four, and six participants, respectively. Participants were assigned to focus groups based on availability. Each group included participants in operational management and leadership roles in quality improvement or care development. The number of focus groups was considered appropriate to address the study’s aim in line with previous research [28], and the discussions generated rich data. A written fictional case scenario (see Additional file 1) was developed based on input from a small group of health and social care professionals with extensive experience collaborating with adults with complex needs. These individuals were not included as participants in this study. The case scenario was used as a starting point for the focus group discussions to facilitate reflection and discussion among participants. Participants were given time to read the case scenario before the interviews began. The discussions were then guided by a semi-structured interview guide (see Additional file 2).

Each focus group interview was conducted by two authors from the research team, and all authors participated in at least one interview. One researcher acted as a moderator and guided the discussion using interview questions and follow-up prompts to encourage reflection and interaction within the group. The second researcher served as the assistant moderator and managed practical aspects, such as monitoring the recording equipment and other logistical matters. Throughout the sessions, the moderator and assistant moderator collaborated to facilitate discussions and ensure a productive interview process.

The interviews lasted 92, 114, and 116 min, respectively, and were audio-recorded and transcribed verbatim by the authors (VP and UH). The participants comprised 13 women aged 41–61 years (missing item data, *n* = 3), with 2–49 years of professional experience working with adults with complex needs (missing item data, *n* = 2). They were employed in municipal (*n* = 9) and regional (*n* = 4) authorities and held positions such as operational managers (*n* = 8) and leadership roles in quality improvement or care development (*n* = 5). Missing data were due to incomplete responses to individual items in the demographic questionnaire on age and professional experience, despite follow-up attempts to obtain the missing information.

Group composition varied across the focus groups: one group comprised one participant from a regional authority and two from municipalities (one operational manager and two quality improvement/care development professionals), another comprised one participant from a regional authority and three from municipalities (two operational managers and two quality improvement/care development professionals), and the remaining group comprised two participants from a regional authority and four from municipalities (five operational managers and one quality improvement/care development professional).

### Data analysis

The data were analysed using qualitative content analysis based on the approach described by Graneheim and Lundman [20, 21]. This method enables a systematic examination of textual data to describe and interpret patterns in participants’ accounts. In this study, the analysis focused on the manifest content of the interviews, that is, the explicit and directly expressed content of the text.

The transcripts were first read several times to gain an overall understanding of the material. The text was then examined in detail to identify meaning units, defined as phrases or sentences related to the study aim. These meaning units were condensed while preserving their core meaning and subsequently labelled with codes reflecting their content. The codes were compared and grouped based on similarities and differences, forming clusters of related content. Through this process, categories representing the manifest content of the data were developed.

The analysis involved continuous movement between individual parts of the text and the entire dataset to ensure consistency and coherence. The initial analysis was conducted by VP in close collaboration with the co-authors (MH and UH). Regular discussions among all authors (MH, VP, UH, and AA) throughout the analytical process supported the development of the categories until consensus was reached, resulting in three categories and eight subcategories. The findings were subsequently interpreted in the Discussion in relation to relevant theoretical frameworks and previous research.

## Results

The data analysis of managers’ experiences working with adults with complex needs resulted in three categories: (1) organizational prerequisites for the sustainable improvement of integrated care, (2) prerequisites for interprofessional collaboration in addressing individuals’ needs, and (3) supporting individual change through relational and needs-based work, with eight subcategories (see Table 1).

**Table 1:** Overview of categories and subcategories

Categories	Subcategories
Organisational prerequisites for sustainable improvement of integrated care	Leadership and governance as prerequisites for changing work practices
	Competence challenges as barriers to address complex needs
	Change management as a shift from quick fixes to long-term learning
Prerequisites for interprofessional collaboration in addressing individuals’ needs	Trust and mutual understanding as prerequisites for collaboration
	Integrated and planned coordination as organisational prerequisites for collaboration
Supporting individual change through relational and needs-based approaches	Individual needs as the basis for change
	Trust-based professional relationships as enablers of change
	Challenging conditions as barriers to individual participation

### Category: organisational prerequisites for sustainable improvement of integrated care

#### Subcategory: leadership and governance as prerequisites for changing work practices

Leadership and governance were described as important prerequisites for changing work practices to improve integrated care for adults with complex needs. Management had key responsibility for establishing support structures and organisational frameworks that facilitated such changes. Governance structures existed in the form of guidelines, routines, and processes; however, these were not always clearly integrated into daily practice. National policy documents and guidelines were sometimes deprioritised in favour of locally developed routines, which hindered the implementation of national directives.

Yes, or, we have sort of set up our own routines, which sometimes counteract care programs, national guidelines. [Participant 2, focus group 2].

To support changes in work practices, participants emphasised the need for leadership to clarify mandates within and between organisations, establish shared guidelines, and promote a stronger focus on individuals’ needs rather than organisational constraints. Leadership and governance were also described as important for supporting changes in work practices by promoting shared responsibility and a mutual understanding of roles and responsibilities across organisations.

#### Subcategory: competence challenges as barriers to address complex needs

Competence-related challenges within organisations were described as barriers to addressing individuals’ complex needs. Limited resources, budget constraints, and recruitment difficulties resulted in insufficient staffing levels, which risked both service quality and the long-term availability of competence. High staff turnover hindered the development of stable teams, professional networks, and ongoing competence development. It reduced continuity in service provision, limited opportunities for the structured introduction of new staff, and complicated communication between professionals, making coordination of care more difficult.

And there is a high turnover of staff; you don’t have time to build up competence, you don’t have time to build relationships with other care providers—that’s important too, because collaboration should be easy. [Participant 1, focus group 1]

Sustainable competence development was described as dependent on stable staffing, structured introduction processes, and a greater understanding of each other’s organisational contexts. It also required shared resources, a more holistic perspective, and organisational support for competence development through cross-sector collaboration, the sharing of successful practices, and the integration of learning into everyday work.

#### Subcategory: change management as a shift from quick fixes to long-term learning

Participants described a need to shift from short-term, reactive solutions towards more systematic and long-term approaches to better meet the needs of adults with complex needs. This shift involved promoting organisational learning, focusing on opportunities rather than obstacles, and sharing experiences from both successful and unsuccessful initiatives across organisations. It also involved discontinuing ineffective initiatives and reallocating resources to support new ways of working collaboratively. Change processes were described as more effective when based on evidence and identified needs and when supported by systematic follow-up to ensure that training and development initiatives were implemented in practice.

Yes, it’s that thing about follow-up, that you stick with it, otherwise you just get a binder to take home and then you have to actually keep at it in the organization and not just come in with a new training later… We push ahead, increase competence, increase competence, but we don’t use the competence. [Participant 4, focus group 1]

Participants described the need for more proactive approaches. Incident management was identified as a way to support shared learning through the collaborative analysis of incidents, particularly when multiple actors were involved in supporting adults with complex needs. However, participants noted that organisations often implemented solutions without conducting thorough root-cause analyses or drawing on lessons from previous experiences. A lack of time for reflection was identified as a key barrier, as urgent situations and heavy workloads were reported to take priority over long-term learning and improvement efforts.

### Category: prerequisites for interprofessional collaboration in meeting individuals’ needs

#### Subcategory: trust and mutual understanding as prerequisites for collaboration

Participants described trust and mutual understanding among professionals and between organisations as important prerequisites to support interprofessional collaboration in the care of adults with complex needs. Structured interprofessional forums were identified as important arenas for collaboration, providing opportunities to build trust and mutual understanding of each other’s competencies, roles, and responsibilities through case discussions, coordination of interventions, alignment of routines, and the development of shared ways of working. Knowledge of each other’s organisational contexts, active relationship-building, and a solution-oriented approach were also described as important for building trust and mutual understanding.

Participants also described challenges in translating discussions during collaboration meetings into concrete actions for the individual. Differences in terminology between organisations could lead to misunderstandings when developing collaboration agreements. In addition, the introduction of digital healthcare systems was perceived to reduce direct, face-to-face communication between professionals across organisations, limiting opportunities for relationship building between professionals.

The more we know each other, the easier and faster it goes because you have trust, and you don’t need all that play around it before you get to where you’re going. [Participant 6, focus group 3]

Collaboration was sometimes described as being overly dependent on individual engagement. To promote more sustainable collaboration, participants emphasised the importance of embedding it within organisational structures through clear roles and formalised assignments, including defined responsibilities in job descriptions and recruitment processes.

#### Subcategory: integrated and planned coordination as organisational prerequisites for collaboration

Participants described the coordination of interventions across organisations as complex and dependent on clear planning and shared structures. When responsibility for different aspects of care was distributed among multiple stakeholders, there was a risk of fragmented interventions and repeated starts in the absence of a coherent plan. Initiatives such as mobile multi-professional teams were reported to facilitate coordination, for example, during transitions from inpatient care to home-based support. However, participants noted that insufficient planning between organisations could lead to long waiting times and interruptions in interventions, which were reported to reduce motivation and increase the risk of relapse. Professionals also expressed hesitancy to initiate collaboration due to concerns about assuming extensive responsibility.

If we pick up the ball, then we get everything, and that’s enough, and we’re not going to sort it out alone, you know. [Participant 1, focus group 3]

Participants described collaboration between the region, municipality, and external actors as essential for addressing complex needs, such as addiction, mental health problems, housing, financial difficulties, and protection needs. As no single organisation was able to address these needs alone, participants emphasised the importance of focusing on an individual’s overall life situation rather than organisational boundaries. At the same time, collaboration was reported to be challenged by the centralisation of agencies, such as the police and employment services, which reduced local presence and limited opportunities for personal contact.

A coordinated individual plan was identified as an important tool for supporting coordinated interventions across healthcare and social services. This was considered particularly important when responsibilities were clearly defined, and meetings were conducted in person, with digital meetings used as complements. Challenges were reported to arise when responsibilities were unclear, when many actors were involved, or when high workloads delayed decision-making. The introduction of a coordinator role was suggested as a way to convene meetings, lead the process, and ensure follow-up.

### Category: supporting individual change through relational and needs-based approaches

#### Subcategory: individual needs as the basis for change

Participants described that interventions needed to be tailored to the individual’s needs to support motivation and lasting change. Individual needs were characterised as dynamic and requiring continuous, long-term assessment, for example, in cases of fluctuating alcohol dependence. Health conditions were reported to influence opportunities for change; new diagnoses were seen to alter treatment options, and psychiatric assessments were identified as necessary to ensure appropriate interventions. Cognitive difficulties related to substance use, such as impaired memory, as well as neuropsychiatric symptoms, including impulsivity and reduced ability to take responsibility, were reported to complicate adherence to agreements between the provider and the individual.

Participants emphasised the importance of structure, routines, and a supportive approach that strengthened individuals’ belief in their ability to change, particularly in unstable life situations. Housing and employment were identified as key factors for stability and long-term change. Social networks were highlighted as central, and network mapping was identified as a useful intervention. Family members were also highlighted as important for sustaining motivation, as professional support was not continuously available.

Participants further emphasised the importance of acting promptly when a ‘window of opportunity’ arose, referring to situations in which individuals were motivated for change and required immediate support. They also noted that staff required sufficient time to identify such opportunities and to support individuals’ capacity for change through supportive interactions. At the same time, participants highlighted the importance of maintaining realistic expectations, as motivation and capacity were reported to fluctuate over time.

It is quite common to have cancellations and the like, or missed appointments, or maybe someone does not show up, depending on where you are in your level of motivation. One day you might want all the help in the world, and the next day you do not want anything. Those are the kinds of shifts that are so difficult in these complex cases, I think. [Participant 2, focus group 1]

#### Subcategory: trust-based professional relationships as enablers of change

Participants described trust-based relationships between professionals and individuals as important enablers of individual change and engagement in interventions. The establishment of such relationships was characterised as a long-term and often challenging process. A relationship-oriented approach, characterised by continuity, regular follow-up, and ongoing learning about the individual, was seen as supporting the development of trust. Building on existing relationships with the individual was also reported to promote stability and increase the likelihood of successful interventions.

Participants emphasised the importance of maintaining contact, even when individuals expressed reluctance or experienced meetings as difficult. Such responses were understood as part of the illness and as requiring persistence from the staff. In the absence of such persistence, individuals who were more ready for change were considered at risk of being prioritised, as they were perceived to have greater prospects for success. At the same time, reluctance to change behaviour was reported to evoke mistrust among staff, which could influence the continuation of support.

The level of professional commitment among staff members varied. Some were described as going beyond formal role expectations to support change, while others maintained clearer boundaries. Participants also expressed a fear of failure, including concerns about exposing individuals to repeated unsuccessful attempts and apprehension about being held responsible in the event of relapse.

And here, with these people, everyone finds it hard to believe in because it’s tough for all of us… And we are exposed, we don’t talk about that, because it’s not only the individual who faces failure, we do too, and that’s why we’re hesitant to trust the relationship next time in some way… No, but neither do we want to put ourselves at risk, if we’re being honest. [Participant 6, focus group 3]

#### Subcategory: challenging conditions as barriers to individual participation

Participants described individual participation as challenging when circumstances limited individuals’ ability or willingness to influence their care and support. Gradually strengthening participation through increased responsibility was viewed as important. For example, within the initiative Mötesplats (a setting where adults with complex needs meet and socialise), individuals were gradually entrusted with responsibilities, such as managing keys and interacting with visitors.

Participation was reported to be more limited during emergency interventions, in which the focus often shifted toward immediate actions rather than the individual’s perspective. Therefore, participants emphasised the importance of first asking about an individual’s wishes before proposing solutions.

You want to solve it, there is a focus on intervention, but you forget about the individual. Thoughts about how you want things to be or how you want to solve them, because there are so many around who are thinking within the context of an emergency situation. [Participant 4, focus group 1]

Participation was also described as challenging when individuals declined interventions or felt compelled to accept them to access services, such as housing. Compulsory care under the Care of Psychiatric Compulsory Care Act (LPT) or the Care of Substance Abusers Act (LVM) meant that voluntariness was set aside. Participants noted that although compulsory care could provide access to treatment, it was sometimes insufficient when appropriate interventions, such as psychological counselling, were not available. Participants described that this lack of appropriate interventions increased the risk of relapse.

Consent was described as a prerequisite for interprofessional collaboration. However, participants noted that obtaining consent could be challenging and required clear, individualised information to ensure that individuals understood what they were consenting to. Coordinated individual plans were highlighted as a tool to support participation by facilitating preparation and enabling clear communication between involved actors. User-controlled admissions within psychiatry, where individuals initiated care themselves, were described as another approach to strengthening participation and increasing individuals’ influence over their care.

## Discussion

The findings contribute to the understanding of how managers perceive and navigate the organisational conditions required to achieve integrated care. From a managerial perspective, the challenges were not primarily related to a lack of awareness of person-centred approaches, but rather to difficulties in translating shared ambitions into sustainable organisational practices. Managers highlighted challenges related to collaboration and individual participation while expressing an ambition to advance more person-centred and integrated approaches.

This is consistent with previous findings from the staff’s perspective [29] and from the Swedish national direction toward integrated care [30]; however, a persistent gap remains between stated goals and practical implementation. While our previous study from the staff’s perspective identified challenges in delivering person-centred and integrated care in everyday practice [29], the managers’ perspective presented here provides additional insight into the organisational conditions shaping these challenges. From the managers’ perspective, translating overarching initiatives into practice requires not only a shared vision but also well-functioning structural, governance, and relational dimensions, as conceptualised by D’Amour et al. [31]. In this context, trust is a central process that develops through a better understanding of each other’s competencies, roles, and ways of working. This reduces uncertainty and enables shared responsibility.

Trust was also identified as a prerequisite for collaboration across professional, organizational, and system levels. This finding reinforces previous research highlighting interprofessional trust as a key component of collaboration [31] and a key factor in how integrated care systems work [16]. However, the managers further highlighted that relational conditions alone are insufficient, and that collaboration is at times dependent on a limited number of highly engaged individuals, creating vulnerability when key actors leave or change roles. In line with D’Amour et al. [31], this demonstrates that sustainable collaboration requires organizational anchoring through clear roles, shared structures, and formalised responsibilities, rather than reliance on individual commitment. Research on inter-organisational collaboration suggests that collaboration across organisational boundaries should be understood as an ongoing process of negotiation, where actors continuously clarify responsibilities, coordinate actions, and construct shared understandings across professional and organisational domains [32]. The present findings suggest that sustainable collaboration depends not only on formal structures and responsibilities, but also on the capacity to facilitate adaptive coordination across organisational boundaries. This perspective is particularly relevant in the context of adults with complex needs, where effective coordination requires organisational adaptability and the capacity to respond to complex and evolving needs across services.

Against the backdrop of extensive system reforms, participants described overload and uncertainty regarding how to manage increasing demands and initiate change processes. Such conditions may hinder the establishment of continuous quality improvement processes that are central to successful implementation, including feedback loops, performance monitoring, and iterative adjustments [33]. Participants’ uncertainty regarding how to approach change processes further points to the importance of theory-informed strategies. Implementation grounded in theoretical frameworks can support the identification and management of underlying behavioural and organizational mechanisms and reduce reliance on ad hoc approaches [34]. In addition, high staff and managerial turnover appears to undermine continuity, limiting the conditions for sustained implementation. This aligns with Isaacs and Mitchell [35] emphasising the importance of standardised workflows, clear role delineation, and outcome measurement in supporting implementation processes.

The findings also highlight the importance of organizational structures that facilitate continuous communication between professionals. From the managers’ perspective, interprofessional forums were described as important arenas for shared decision-making and the development of common practices. Previous research [9, 12, 36] has emphasised that regular meeting structures and shared arenas for dialogue can strengthen coordination and promote shared understanding between professionals. Co-location was also identified as a facilitating factor, enabling informal communication and coordination, which is consistent with evidence suggesting that integrated service settings can improve coordination and access to care [35, 37].

In addition to organizational arrangements, governance and leadership appear to play a critical role in enabling sustainable collaboration. Leadership can provide direction and legitimacy for interprofessional work practices [31, 38]. However, the findings indicate that integrated care is often insufficiently prioritised and resourced, with expectations that collaboration should be incorporated into existing structures. This reflects previous research showing that unclear governance, ambiguous responsibilities, and limited resources can hinder implementation and contribute to unequal access to integrated care [16, 38]. According to Oelke et al. [39], policy and governance are therefore central to shaping how collaborative and team-based models are realised in practice.

Finally, managers described collaboration as needing to be organised around the individual’s needs and life situation. Relational continuity, regular follow-up, and a deeper understanding of the individual were described by managers as essential for supporting adults with complex needs. This aligns with Czypionka et al. [12] highlighting the importance of patient involvement in care planning and goal setting within integrated care. The need for a designated contact person or care coordinator also emerged, reflecting previous findings from Harris et al. [29] that such roles can support navigation across fragmented systems and foster continuity. Managers perceived trust-based relationships between professionals and individuals as central to enabling person-centred care. Although these findings concern processes close to the individual level, managers described them from an organisational perspective, highlighting the need to create structures that enable continuity, coordination, and person-centred approaches.

While previous research has largely explored integrated care from the perspectives of patients and frontline professionals, this study contributes by highlighting how managers perceive the organisational prerequisites for achieving integrated care. The findings illustrate how managers experience the challenges of translating strategic intentions into everyday practices through organisational structures, resource allocation, leadership, and ongoing negotiation across organisational boundaries.

### Methodological considerations

Trustworthiness was addressed in line with Lincoln and Guba’s [40] criteria of credibility, dependability, confirmability, and transferability. Credibility was strengthened through purposeful sampling of managers with relevant experience of working with adults with complex needs in healthcare and social services. The variation across organisational contexts and professional experience constitutes a strength, as it enabled the inclusion of diverse perspectives.

The use of a case scenario provided a structured starting point for the focus group discussions and facilitated meaningful reflection among participants by offering a shared basis for discussion. In addition, three focus group interviews generated detailed and relevant data, and the composition of the groups supported nuanced discussions of integrated care across organisational boundaries. Although two of the focus groups included relatively few participants, this may have limited the breadth of perspectives represented and the interaction between participants. Nevertheless, these groups generated rich and relevant discussions.

Dependability was supported by a systematic and transparent analytical process, as described in the Methods section. Confirmability was strengthened through ongoing dialogue within the research team, supporting reflexivity and ensuring that the findings were grounded in the data. The use of participant quotations further enhanced transparency and confirmability.

Transferability was supported by providing detailed descriptions of the study context, participants, and data collection procedures. However, all participants were women, which reflects the gender distribution in healthcare and social services in Sweden, where women are overrepresented, including in managerial roles [41]. This may affect transferability, as perspectives from men are lacking. Furthermore, as the study was conducted in one Swedish region, the transferability of the findings to other contexts should be considered with caution. However, the inclusion of managers from both municipal and regional organisations, representing different leadership roles, may enhance the transferability of the findings to similar organisational contexts. The focus group design may also have limited the expression of individual or critical views [19] as participants held managerial responsibilities across organisations and processes. Nevertheless, both the moderator and assistant moderator experienced the discussions as open, with participants reflecting on challenges related to collaboration across organisational boundaries.

## Conclusions

From the managers’ perspective, interprofessional collaboration in the care of adults with complex needs is influenced by relational, structural, and contextual factors. Although managers express awareness of and ambition towards person-centred and integrated care, a gap remains between intention and implementation. Strengthening and sustaining trust, alongside clearer structures, governance, and organisational continuity, may support more sustainable collaboration by creating organisational conditions that enable individual participation.

Challenging organisational conditions, such as a high workload and staff turnover, may limit the capacity to implement and sustain systematic processes, including continuous quality improvement. Strengthening implementation capacity through more structured, theory-informed approaches, while supporting organisational conditions that enable continuity, may be important for translating collaborative ambitions into sustained practice. Further research is needed to explore how integrated healthcare and social services can be effectively implemented and sustained for adults with complex needs.

## Supplementary Information

Below is the link to the electronic supplementary material.

Additional file 1. Patient case

Additional file 2. A semi-structured interview guide

## Data Availability

Data excerpts are provided in the manuscript. The qualitative data collected in this study are not publicly accessible because of the challenges in ensuring complete anonymity. The participants did not provide consent for public sharing of their data. The datasets at an aggregated level are available from the corresponding author upon reasonable request.

## Declarations

### Author’s information

The research team has complementary expertise in qualitative research, nursing, leadership, and research and development. All authors have experience of qualitative content analysis, qualitive interviews and scientific publishing.
