# Supplementary material for: Managers’ experiences of collaboration and individual participation in integrated healthcare and social services for adults with complex needs: a focus group study
Source: BMC Health Serv Res. 2026 Sep 12;26:1273. doi: 10.1186/s12913-026-15602-y (PMC13579995; doi:10.1186/s12913-026-15602-y)
Supplement: Supplementary file 1 [file 12913_2026_15602_MOESM1_ESM.docx]

**Case**

The individual is in their early 30s and presents with a long-standing history of polysubstance use beginning in adolescence. Contact with social services has occurred intermittently over time, primarily in relation to ongoing substance use. There is a suspected psychotic disorder; however, the individual seeks only pharmacological treatment for ADHD and declines further psychiatric involvement.

The clinical history includes approximately 20 admissions to residential treatment for substance use, as well as multiple contacts with psychiatric intensive care units and emergency psychiatric services. Previous care episodes have been complicated by behavioral issues, including violence toward others and an incident involving assault on staff at a treatment facility, which led to discharge.

The individual’s social situation is unstable. Housing has been inconsistent, with repeated relocations between municipalities and periods spent outside the region. The individual has experienced eviction and homelessness, and temporary residence with the mother is no longer possible. At present, the primary request is assistance in obtaining housing in another municipality. There is also an ongoing threat related to criminal networks due to outstanding debts. A legal guardian has been appointed, with responsibility limited to financial matters.

The most recent contact with the health center was prompted by deteriorating mental health and resulted in referral to the emergency department. Inpatient care was offered but declined, as the individual requested only ADHD medication. Assessment at that time concluded that the criteria for compulsory psychiatric care were not met. Due to continued substance use, a notification of concern was submitted to the municipality under Section 6 of the Care of Substance Abusers Act (LVM). The individual did not initially express a need for support; however, social services subsequently initiated contact. The individual later applied for assistance with housing and support in accessing ADHD medication and consented to a Coordinated Individual Plan.
