# Supplementary material for: Managers’ experiences of collaboration and individual participation in integrated healthcare and social services for adults with complex needs: a focus group study
Source: BMC Health Serv Res. 2026 Sep 12;26:1273. doi: 10.1186/s12913-026-15602-y (PMC13579995; doi:10.1186/s12913-026-15602-y)
Supplement: Supplementary file 2 [file 12913_2026_15602_MOESM2_ESM.docx]

**A semi-structured interview guide**

Participants receive information about the format and procedures of the focus group interviews. At the start of the session, they are asked to read a fictional case and reflect on it individually for a few minutes.

The interview starts with the open-ended question, please tell me what your thoughts on this case are.

**Follow-up questions that can be used when needed**

How would you handle this situation based on your professional role?

How are similar situations usually handled in practice, and how do you think they should be handled? Why?

What possible courses of action do you identify and what opportunities and risks are associated with them?

What are the key challenges and strengths in this situation?

How do you understand the concept of participation in your work?

How can individual participation be promoted in this case, and what factors may facilitate or hinder it?

What working methods or organizational structures are currently in place to support participation?

How can you, in your leadership role, create or strengthen the conditions for participation?

How does collaboration function for adults with complex needs, both internally and externally?

What factors facilitate or hinder collaboration?

How do regulations, division of responsibilities, or organizational boundaries affect your work?

Do you perceive a shared understanding of working approaches among the actors?

Could you describe your knowledge of each other’s roles and responsibilities?

Do you perceive that you have sufficient knowledge and support to lead and develop work for adults with complex needs?

What support is available and what is lacking?

Can you provide an example of a situation in which the work functioned particularly well?

What factors contributed to this, and what lessons can be learned?

Follow-up questions, such as “Could you give an example?” and “Can you elaborate on that?”, will be asked as needed throughout the interview to further explore and deepen the topics that emerge.
